# *Diospyros villosa* Root Monographic Quality Studies

**DOI:** 10.3390/plants11243506

**Published:** 2022-12-14

**Authors:** Adriana Ribeiro, Rita Serrano, Isabel B. Moreira da Silva, Elsa T. Gomes, João F. Pinto, Olga Silva

**Affiliations:** Research Institute for Medicines (iMed.ULisboa), Faculty of Pharmacy, Universidade de Lisboa, 1649-003 Lisboa, Portugal

**Keywords:** botanical characterization, chemical identification, *Diospyros villosa*, herbal medicines, hydrolyzable tannins, histochemistry, microscopy, polyphenols, quality control, toothbrush

## Abstract

*Diospyros villosa* L. (De Winter) (*Ebenaceae*) is a shrub whose root (DVR) is used as a toothbrush and to treat oral infections in Mozambique. The present work aims at establishing monographic quality criteria to allow the sustainable and safe development of pharmaceutical preparations with this herbal drug. This includes setting botanical (qualitative and quantitative) and chemical identification parameters, purity tests (loss on drying and total ash), quantifying the major classes of constituents identified, and particle size characterization of the powdered drug. DVR samples are cylindrical and microscopically characterized by: a periderm, with six layers of flattened phellem cells, with slightly thickened walls and few layers of phelloderm; cortical parenchyma with brachysclereids with a short, roughly isodiametric form (13.82–442.14 μm^2^ × 10^3^), surrounded by a ring of prismatic calcium oxalate crystals; uniseriate medullary rays and prominent vessels of the xylem with single or double shape; numerous single and clustered starch grains, within the cortical parenchyma, medullar parenchyma, and ray cells. Polyphenols, mainly hydrolyzable tannins (212.29 ± 0.005 mg gallic acid equivalent/g of dried DVR), are the main marker class of constituents. Furthermore, the average diameter of the particles of the powder, 0.255 mm, allows its classification as a fine powder.

## 1. Introduction

*Diospyros villosa* L. (De Winter) is a shrub belonging to the *Ebenaceae* family, the root of which has been used for many years to treat oral infections, namely as a toothbrush [1,2], particularly in Mozambique where it is native, occurring naturally in the coastal grasslands [3,4].

The use of the family *Ebenaceae* medicinal plants is common and remarkable for treating oral diseases [5,6,7]. Similarly to *Diospyros villosa*, the branches and roots of *Euclea natalensis* A. DC., also an *Ebenaceae*, are traditionally used for chewing, as sticks for tooth cleaning, and as a mouthwash for treating toothaches [8]. In traditional African medicine, *D. villosa* root is also a traditional herbal medicine to treat digestive and musculoskeletal complaints [9,10,11].

The ethnomedical use of the *Diospyros* L. genus has distinct and important impacts on treating various diseases, namely malaria and opportunistic diseases associated with HIV/AIDS [6,7,12,13]. In addition, different pharmacological properties have been reported for species of this genus, such as antimalarial, analgesic and anti-inflammatory, antibacterial, and gastroprotective properties [14,15,16,17].

The most representative classes of compounds isolated from the *Diospyros* genus were 1,4-naphthoquinones [18,19], triterpenoids (especially lupane, ursane, oleanane, and taraxarane derivatives), benzopyrones, and polyphenols [20,21], which showed free radicals’ inhibitory activity [22]. Other chemical classes of compounds, namely hydrocarbons, lipids, amino acids, sugars, and tetraterpenoids (carotenoids), were also identified [23].

The Mozambican population commonly recognizes *Diospyros villosa* as hairy star-apple and shaggy diospyros (English), harige sterappel (Afrikaans), or muhangula (Chopi) in the Gaza region, and “chicombela” or “mulala,” a vernacular name common for *Euclea natalensis*, in the Maputo geographic region [4]. The roots are the most common plant part of these species as medicinal plants, and their medicinal use is sometimes applied undifferentiated. However, *Euclea natalensis* root has already been the subject of some chemical, pharmacological, and pharmaceutical development research [8,24,25,26]. 

**Figure 1:**
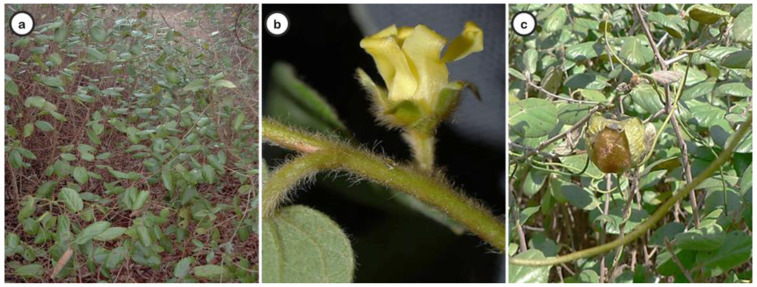
*D. villosa* in nature (**a**); detail of flower (**b**) and fruits (**c**). Photography by: Olga Silva & Elsa Gomes.

*D. villosa* (Figure 1) constitutes a shrub erect or scandent shrub 1–4 m high (sometimes higher) or a rhizomatous subshrub up to 0.5 m tall, or, alternatively, a small tree [4]. The leaves are thin and rigid (Figure 1a), the flowers are dioecious, and the female is usually more prominent than the male (Figure 1b). The fruits, up to 3 × 3.8 cm, are globose, depressed, and dehiscent (Figure 1c). Seeds 3–8, up to 1.5 cm long, are opaque brown with a soft endosperm. The plant is a diploid species (2*n* = 2 × 30 chromosomes) [27,28].

The characterization of *D. villosa* dried leaf through microscopic techniques was performed by Gromek et al. (2011), and, recently, his study was complemented by Adu et al. (2022), together with the microscopic characterization of the stem bark of this species [29,30]. Additionally, the determination of the antimicrobial activity and toxicological evaluation of a leaf ethanolic extract of *D. villosa* dried leaf was already performed in our lab [31].

Previous studies conducted by Cirera [32] and Cirera et al. [33] on *D. villosa* root confirmed the in vitro antimicrobial activity of a 70% hydroethanolic extract of this medicinal plant against different microorganisms, including the oral bacteria *Lactobacillus plantarum*, *Lactobacillus rhamnosus*, *Lactobacillus delbruecki* subsp*. bulgaris*, and *Lactobacillus bulgaris*, which showed this extract to be especially active against *Escherichia coli*, *Enterococcus faecalis*, *Staphylococcus aureus*, and *L. plantarum*. These results agreed with the traditional use, described above, of this medicinal plant, and chemical studies carried out allowed the identification of polyphenols, naphthoquinones, and triterpenoids as the main classes of constituents present, and among these, the identification of a gallic acid derivative and beta-sitosterol as marker constituents [2,32]. 

The evaluation of the in vivo acute toxicity by administration of the upper cited *D. villosa* root extract allowed the verification of a possible renal dysfunction development in mice. These results agree with the possible safe topical administration of this herbal preparation but not with its oral administration [32].

The root, dried and fragmentized, is the main *D. villosa* plant part sold in the local markets [32]. As its medicinal benefits have been confirmed, it is now essential to increase its study, aiming at the aspects of quality, safety, and efficacy, to be considered in the future, as a herbal drug or substance according to the requirements of the European Medicines Agency (EMA) for modern medicines [34]. Given that, currently, a medicinal plant is considered the active ingredient of a herbal drug or substance, and according to the European Pharmacopoeia, assessing its quality includes the identification Tests-knowledge of its macromorphological characteristics (Test A) and a microscopic of the powdered herbal raw material samples (Test B), as well as the knowledge of its chemical profile, established by thin layer chromatography (TLC) or High-performance TLC (HPTLC), which aims not at the structural identification of marker compounds of this medicinal plant but rather at the characterization of a set of constituents of a particular chemical class, and which may or may not be involved in the herbal drug biological activity (Test C); Purity Tests-determination of moisture content, ash, foreign elements, etc. In addition, Dosing-Mostly spectrophotometry aims at marker compound classes, expressed in a given compound of this class, and not at a concrete compound [35].

For *D. villosa* root, the results of a previous study aiming at the morphological characterization of the sample used by Cirera et al. in their studies [32], allowed the establishment of some morphoanatomical criteria useful for the botanical diagnosis of this medicinal plant as a herbal drug. However, to better characterize *D. villosa* root as raw plant material for pharmaceutical use, complementary studies are considered necessary to improve and confirm the monographic criteria already described, using a larger number of samples, from at least one different origin, and performing deeper botanical microscopic qualitative and quantitative analysis. In sequence, this identity must be confirmed through the chromatographic profile by TLC and or HPTLC. The monographic elements relating to purity and assay are also necessary to allow the development of standardized traditional preparations and of final pharmaceutical forms of them, capable of use for therapeutic purposes after the confirmation of their efficacy and safety. The results of the additional botanical work that have been identified as necessary (qualitative and quantitative and histochemical) and of the chemical identification setting parameters (TLC fingerprint establishment), purity tests (loss on drying and total ash), and quantification of the main marker compounds will be presented and discussed. Additionally, the *D. villosa* root powdered particle size was also characterized to ensure resampling and reproducibility of the pharmaceutical preparation methods involving this medicinal plant.

## 2. Results

### 2.1. Botanical Studies

#### 2.1.1. Macroscopic Analysis

Macroscopically, *D. villosa* root samples are cylindrical, generally linear or, more rarely, branched, presented in fragments of 9–21 cm in length and 2–11.5 cm in diameter (Table 1).

**Figure 2:**
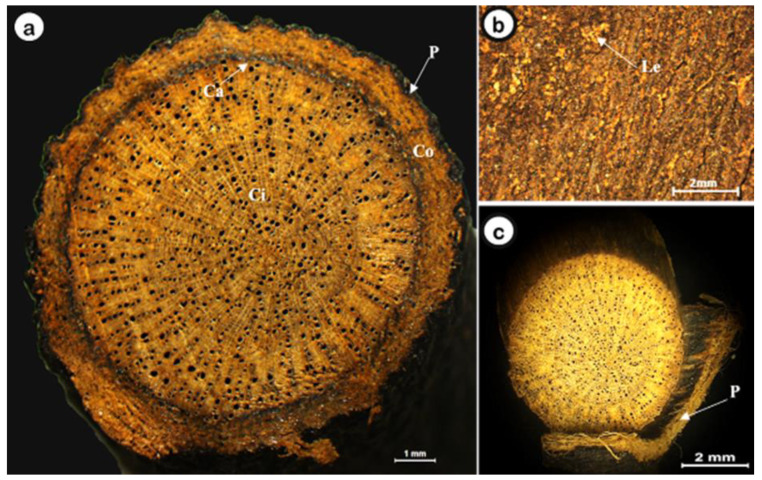
General aspects of *D. villosa* root. (**a**): Transverse section with, Periderm-P, Cortex-Co, Cambium-Ca, Central Cylinder-Ci; (**b**): Lenticels; (**c**): Peridermis highlighted. Bars: **a** = 1 mm; **b** and **c** = 2 mm.

The *D. villosa* root cross-section (Figure 2) shows four distinct regions: the periderm, the cortex, the cambial “band” and the central cylinder (Figure 2a). The cortex is yellowish-brown, and the central cylinder is light yellow with a fine radial striation, occupying about three-quarters of the root diameter. Traces of secondary roots or mainly rounded scars from their fall may occur. Lenticels are frequently observed (Figure 2b). The periderm may be partially missing, and the inner layers, which are yellowish-brown, can be seen in these faults (Figure 2c).

#### 2.1.2. Microscopic Analysis

**Figure 3:**
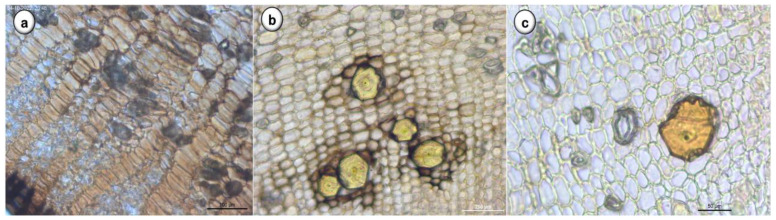
Transverse section of *D. villosa* root. (**a**–**c**): General aspects of cortical parenchyma (**a**): Groups of sclereids; (**b**,**c**): brachysclereids and sclereids; Bars: **a** = 100 µm; **b** = 200 µm; **c** = 100 µm.

Using light microscopy, in transversal view (Figure 3), *D. villosa* root is characterized by groups of sclereids and medullary rays from the central zone (Figure 3a), cortical parenchyma cells with brachysclereids and sclereids in groups (Figure 3b) and one isolated brachysclereid (Figure 3c) in the cortical zone. 

**Figure 4:**
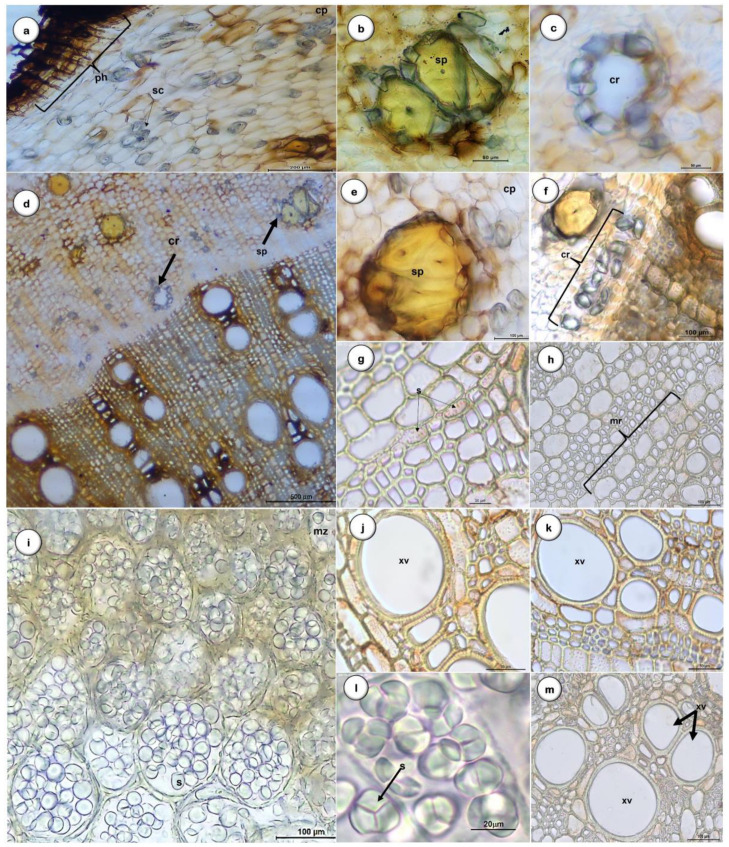
Transverse section of *D. villosa* root. (**a**): General aspects of periderm and cortical parenchyma, peripheral zone detail with phellem, phellogen, and phelloderm; (**b**–**f**): View of the cortical parenchyma and the separation zone of the vascular cambium between the secondary phloem and secondary xylem; (**b**–**f**): Cortical parenchyma cells with isolated brachysclereids and sclereids in groups; (**f**): Brachysclereids with a ring of prismatic calcium oxalate crystals; (**g**,**h**): Xylem and medullary rays uniseriate; (**i**–**l**): Medulla with storage parenchyma and starch grain detail with y-shaped hilum; (**j**,**k**,**m**): Detail of single or double xylem vessels. Bars: **a** = 200 µm; **b**, **c**, **j**, and **k** = 50 µm; **d** = 500 µm; **e**, **f**, **h** and **m** = 100 µm; **g** and **l** = 20 µm. Abbreviations: cp, cortex parenchyma; dr, druse calcium oxalate; cr, prismatic calcium oxalate crystals; mr, medullary rays; mz, medulla zone; ph, phloem; pz, periderm zone; s, starch grain; sc, stone cells; sp, polygonal sclereids; xv, xylem vessels.

In addition, in transversal view (Figure 4), it is possible to observe that the periderm is visible in the outermost part of the root, with a radial arrangement consisting of the phellem, a most pigmented homogeneous tissue with approximately six layers of suberized cells with thicker cell walls, and the phelloderm with cells presenting thin cell walls (Figure 4a). 

The cortex comprises rounded cells, occasionally thin-walled polygons, and several types of sclerenchyma cells with thick and lignified walls (sclereids), frequently in groups of 4 to 10 with an oval to elongated shape (Figure 4e). Brachysclereids of different sizes are the most abundant type of sclereids (Figure 4b–f) and are characterized by an isodiametric or an oval to elongated shape; they have a very thick cell wall and a small central spot corresponding to a reduced protoplasm (Figure 4b–f) and are surrounded by prismatic calcium oxalate crystals (Figure 4f).

Several layers of cells characterize the cortical parenchyma with non-lignified walls and numerous starch grains, such as those present in the medullary parenchyma (Figure 4i). These starch grains, evidenced by the iodine solution, can be small and numerous or bulky, round, or ovoid in shape, with the central region in the form of a ‘y’ or a ‘star’ (Figure 4l). 

The phloem is significantly reduced, and the secondary xylem has a radial arrangement (Figure 4g,h). Single or double xylem vessels with areolate pits on the cell wall (Figure 4j,k,m) and fibers arranged in uni- or bi-seriate radial series, alternated with uniseriate medullary rays, are observed. The primary xylem occupies a medullary position.

**Figure 5:**
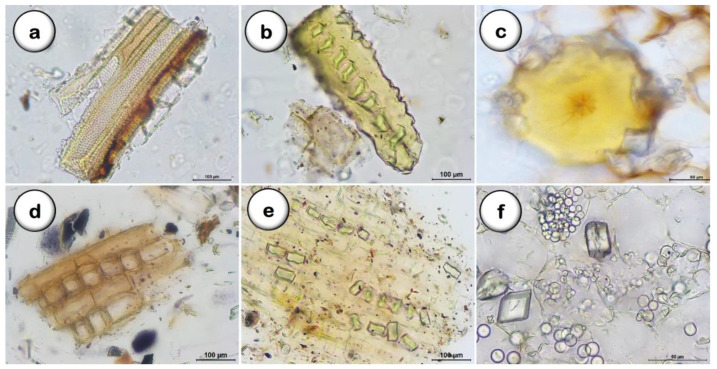
*D. villosa* root, powder. (**a**): Xylem vessels associated with fibers and medullary rays; (**b**): Libriform fibers; (**c**): Brachysclereid with a ring of prismatic calcium oxalate crystals; (**d**): uniseriate medullary rays; (**e**,**f**): Starch grains and prismatic calcium oxalate crystals. Bars: **a**, **b**, **d** and **e** = 100 µm; **c** and **f** = 50 µm.

Following grinding, the microscopical observation of the powdered *D. villosa* root (Figure 5) allows the statement of the main structures previously observed in the cross-sections cuts, such as xylem vessels associated with fibers and with pits areolate (Figure 5a); libriform fibers with prismatic calcium oxalate crystals (Figure 5b); brachysclereids surrounded by a prismatic calcium oxalate crystal layer (Figure 5c); uniseriate medullary rays (Figure 5d); isolated starch grains and prismatic calcium oxalate crystals (Figure 5e,f).

#### 2.1.3. Quantitative Microscopy

The results of the quantitative analysis of the *D. villosa* root microscopic morphological features are presented in Table 2.

#### 2.1.4. Histochemical Tests

**Figure 6:**
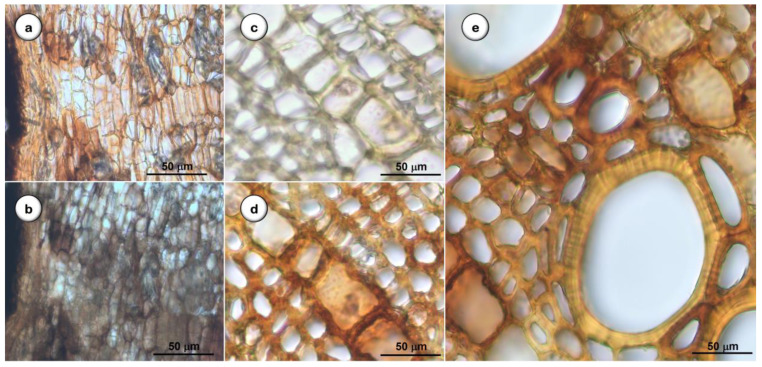
*D. villosa* root, histochemical characterization. (**a**–**c**) before, (**b**) quinones, and (**d**,**e**) tannins. Bars = 50 µm.

Results of the histochemical analysis (*n* = 30) made on the cross sections of the *D. villosa* root are presented in Figure 6 and Table 3: the presence of starch grains among medullary rays, parenchyma cells, and medullar parenchyma was confirmed (Figure 6b); quinones derivatives were detected in the cortical parenchyma cells and medullar rays (Figure 6b); and polyphenols (tannins) were detected in the cortical parenchyma cells (Figure 6d,e). The presence and identification of the calcium oxalate crystals was confirmed using the hydrochloric acid (10%) dissolution test. Lipids and mucilage were not detected in the samples.

### 2.2. Chemical Studies

#### 2.2.1. Extraction

The 70% hydroethanolic extract obtained from the dried powder of *D. villosa* root (particles with a size range between 0.053 mm and 1.086 mm, and a geometric mean size of 0.249 mm) presented a drug-extract ratio (DER) of 6.35:1 (*w*/*w*).

#### 2.2.2. Classification of Particle Size on Powdered Plant Material

Based on the experimental results (Table 4), the interquartile dispersion (IQR) value was determined to equal 0.435 mm, while the geometric mean value was 0.249 mm. These results showed that the *D. villosa* root powder granulometry corresponds to an average value (D50) of 0.255 mm.

Effect of particle size on extract yield: The yield obtained for the different particle sizes (45 μm, 0.355 mm, and 1000 mm) and for the total composition (TC) of the powder (0.053 mm to 1.086 mm) of *D. villosa* root powdered (Table 5) is presented in Table 5. 

The TC *D. villosa* root powder, with an average value of 0.255 mm, gives approximately 15.7% dry powder weight, the highest extract yield. Therefore, this powder was considered the most adequate for the *D. villosa* root extract preparation and chemical studies prosecution (thin-layer chromatography profile and major classes of secondary metabolites quantification).

#### 2.2.3. Thin-layer Chromatography (TLC)

The *D. villosa* root chromatographic fingerprint obtained by TLC of the selected 70% hydroethanolic extract is characterized by the presence of two major bands, one with a purple-bluish fluorescence with an R_f_ of 0.40, corresponding to a gallic acid derivative and band B, possessing a blue-green fluorescence with an R_f_ of 0.60, compatible with the presence of a hexahydroxydiphenolic acid derivative, based on the chromatographic and spectral characteristics described by Cirera et al. for these kinds of compounds [33].

#### 2.2.4. Quantification of Secondary Metabolites by Spectrophotometry

The results of the quantification of the major classes of secondary metabolites identified by TLC (phenol derivatives including hydrolyzable tannins) on the selected *D. villosa* root 70% hydroethanolic extract are presented in Table 6.

These results clearly show that the hydrolysable tannins (with a content of 212.29 ± 0.005 mg GAE/g) were the main class of metabolites in the *D. villosa* root extract.

#### 2.2.5. Analysis of Loss on Drying and Total Ash

The *D. villosa* root obtained values for the loss on drying, and total ash content was 8.40 ± 0.13% and 1.93 ± 0.04%, respectively.

## 3. Discussion

To better characterize *D. villosa* root as raw plant material for pharmaceutical use, complementary studies were made. The obtained results lead us to confirm and improve the botanical quality specifications required to improve and confirm the monographic criteria for identifying this part of the plant as a herbal drug. Morphologically-observed *D. villosa* root dried samples showed a diameter range of 2–11.5 cm, a black to dark brown outer surface longitudinally wrinkled, and inner layers with a yellowish color [36]. The microscopic markers for *D. villosa* root identification include the periderm, composed of six layers of flattened phellem cells with slightly thickened walls and a few layers of phelloderm. The cortical parenchyma has several layers of cells with brachysclereids with a roughly isodiametric shape and a ring of prismatic calcium oxalate crystals. Additionally, there are groups of between four and ten sclereids, phloem crossed by uniseriate medullary rays, and prominent vessels of the xylem occurring in single or double shapes, with bordered holed vessel walls associated with fibers.

Amongst the most characteristic structures of the *D. villosa* root powdered drug are fragments showing libriform fibers with calcium oxalate crystals and numerous single and clustered starch grains within the cortical parenchyma. Additionally, we can observe uniseriate medullary rays, medullar parenchyma, and brachysclereids with a ring of prismatic calcium oxalate crystals. The root hairs were not detected in our samples, although trichomes have been reported to be present in the aerial part of this species [29,30].

The histochemical analysis of *D. villosa* root samples allowed the identification of phenol derivatives as main secondary metabolites (polyphenols and quinones) and starch. Terpenoids, a significant class of constituents referred to in previous phytochemical studies conducted by our team, seem to be minor compounds in our samples [37]. However, alkaloids were not detected in our samples, although this chemical class has recently been described as present in this species’ leaf and stem bark [30].

Antimicrobial properties involving the inhibition of extracellular enzymes, oxidative phosphorylation, or deprivation of substrates necessary for microbial growth were attributed to phenolic compounds, namely tannins [38]. Furthermore, the identification of the presence of these compounds may be correlated with the anti-inflammatory and antibacterial properties that have been reported to this medicinal plant [17].

The granulometric distribution of extract particles is fundamental to guarantee the reproducibility of the tests carried out, with the classification of this distribution being of paramount importance for the resampling or re-extraction of components from the corresponding plant material [39]. Thus, for *D. villosa* root, a maximum extract yield of approximately 15.7% of dry powder weight was obtained, a better result than the one obtained early by our team (around 11.87%). Taking into consideration that various factors, such as cultivation and collection conditions, among others, influence the production of secondary metabolites in a plant, this difference can be seen as acceptable, especially considering that a granulometric control study was not performed beforehand [37]. Regarding the DER (6:35:1 to 8:00:1) obtained using different *D. villosa* root powdered particle sizes, for a maximum yield of the dried extract with the 1 mm particles, an excess of 1.65 g of the dried plant was needed. This aspect is particularly relevant when considering large-scale production, where a significant increase in plant material would be required. In addition, this optimized control is essential for further work on plant materials regarding the required quality control of the plant material as a raw material to produce medicines. According to the European Pharmacopoeia 10, the obtained values for the average diameter of the powder particles are 0.255 mm, allowing its Classification as a fine powder [40]. 

The results of the chemical characterization tests showed that the phenolic compounds, particularly the hydrolysable tannins, are the main class of chemical marker constituents of the prepared extracts. The polyphenolic chemical profile of *D. villosa* root, established by TLC, agrees with the high content of total phenols and hydrolysable tannins determined. In fact, the chromatographic characteristics of band B (R_f_ = 0.60, dark blue fluorescence) are similar to the ones obtained with gallic acid (R_f_ of 0.59) used as a standard. This similarity suggests that this major compound can be a gallic acid derivative [41].

According to the European Pharmacopoeia 10.0, concerning the purity evaluation, two fundamental determinations are the loss on drying and the total ash content as they allow the knowledge of the samples’ water and minerals content. These parameters were determined in our *D. villosa* root samples and can be used as references for the analyses of other samples from the same medicinal plant [42,43].

## 4. Materials and Methods

### 4.1. Materials

Gallic acid standard was provided by Sigma-Aldrich^®^ (Steinheim, Germany). While the ethanol was provided by Aga^®^ (Prior Velho, Portugal), the Glacial acetic acid, acetone, Folin-Ciocalteu reagent, potassium iodate GR, sodium carbonate, and Thin layer chromatography (TLC) cellulose plates were provided by Merck^®^ (Darmstadt, Germany). All of the reagents used were of analytical grade.

### 4.2. Botanical Material

The roots of *Diospyros villosa* L. (De Winter) were collected by E. T. Gomes in the Southern region of Mozambique (278 Km North of Maputo, 10 Km from Madender, in 2019). The voucher specimens of the botanical material were deposited at the LISC-Herbarium, Tropical Botanical Garden of “Instituto de Investigação Científica Tropical”, and the identification was made by the collector in comparison with the voucher samples from the author collection, with the code “O. Silva, s.n. 15.10.2006”. The roots were dried at room temperature, in the absence of direct light, according to the standard techniques of the European Pharmacopoeia 10.0 [35] and stored at room temperature, protected from light.

The correct scientific name of the plant was verified in the “*The Plant List*” http://www.theplantlist.com (accessed on 20 September 2022) [44] corresponding to WCSP (World Checklist of Selected Plants (data supplied on 23 March 2012), accessed in 2019, and currently it has been confirmed on “WFO (2022): World Flora Online” available at the website http://www.worldfloraonline.org (accessed on 14 November 2022) [45].

### 4.3. Botanical Studies Methods

#### 4.3.1. Macroscopic Analysis

The plant material samples were directly examined by the naked eye and by microscopy (Olympus SZ61 Stereo Microscope, Switzerland), coupled with a digital camera (Leica MC170 HD) controlled by proprietary software [Leica Application Suite (LAS) Version 4.8.0, Heerbrugg, Switzerland].

#### 4.3.2. Light Microscopy

The plant material samples (*n* = 30) were submerged in glycerin 30% to soften the surfaces and allow the sections to be sliced. For anatomical analyses, transverse and tangential longitudinal sections were manually prepared (approximately 1 mm thick), cleared and mounted in a 60% chloral hydrate aqueous solution and examined under the microscope (Olympus CX31, Switzerland) coupled with a digital camera (Leica MC170 HD) controlled by property software (Leica Application Suite, version 4.8.0, Switzerland). A representative portion of the total collected raw material selected to study was powdered using an Analytical Mill A-10 water-cooled laboratory mill (Staufen, Germany) and mounted in a 60% chloral hydrate aqueous solution (European Pharmacopoeia 10.0 [46]).

#### 4.3.3. Histochemical Tests

Histochemical analyses were performed in different root sections, using Lugol’s solution for starch detection; Borntraeger reaction for naphthoquinones detection; Sudan Black III for lipids detection; ruthenium red for mucilage detection; potassium dichromate for polyphenols detection; and 2,4-dinitrophenylhydrazine, sulphuric anisaldehyde and Liebermann-Burchard test for terpenes detection [47]. Control tests were performed simultaneously, and the results were observed by light microscopy.

#### 4.3.4. Quantitative Analysis

The macroscopic features were identified by direct observation of the samples. Different samples (*n* = 30) were considered (*n* = 10). The microscopic evaluation was made on 1 mm^2^ of each sample (*n* = 30) in triplicate (European Pharmacopoeia 10.0 [46]). The dimensions and number of the anatomical features selected were determined using the Leica Application Suite (version 4.8.0, Switzerland) software.

### 4.4. Chemical Studies Methods

#### 4.4.1. Extraction

The *D. villosa* root hydroethanol extract (EtOH-H2O, 70:30 *v*/*v*) was prepared by maceration at room temperature (T = 22 °C), under agitation during 24 h, until exhaustion (seven consecutive extractions) in a proportion of 10 L/Kg (1:10). The extract obtained was vacuum filtered using a Glass Filter G4 and a water pump Yamato WP-25. The filtrate was concentrated under pressure using a rotative evaporator (Buchi R-210) at a temperature of less than 35 °C and freeze-dried (T = −57 °C) using a Heto LyoLab 3000 apparatus. Then, the dried extract was stored in a desiccator at room temperature and protected from light, until it reached constant weight, prior to storage in amber glass vials until further use.

#### 4.4.2. Classification of Particle Size on Powdered Plant Material

To prepare the extract, the plant material was ground into a powder with a mill (IKA MultiDrive BL200 mill) to decrease the particle size. Tests (*n* = 3) were carried out with approximately 100 g of pulverized plant material, which was sieved using a set of sieves with a mesh opening between 0.053 mm and 1.086 mm, with increments of √2, according to ISO 3310-1 (Retsch, Haan, Germany). After 15 min of agitation with a medium vibration amplitude (AS 200 digits, Retsch, Haan, Germany), the amount of powder that was retained on each sieve was checked to allow the determination of the geometric mean size for the population of particles. In sequence, the geometric mean of the particle size for each sieve was determined. Based on the values obtained, the D50 (median), D25, and D75 (quartiles) were calculated, allowing the calculation of the interquartile (D75–D25). Powdered root particles were split in different particle size ranges, namely 45μm, 355 mm, 1000 mm, and total non-sieved samples (TC) of the powdered *D. villosa* root (0.053 mm to 1.086 mm), to evaluate the effect of particle size on extract yield: sieved samples were subjected to ethanol extraction, as described in Section 4.4.1.

#### 4.4.3. Thin-Layer Chromatography (TLC)

The chemical profile of *D. villosa* root was established by TLC, using different hydro-ethanol extracts of *D. villosa* root. Gallic acid (GA) was used as a standard, the TLC cellulose plates (Merck^®^) as the stationary phase, and acetic acid-water (2:8, *v*/*v*) as the mobile phase [47]. The sample was spotted using 10 µL micropipettes, and each plate was derivatized by the NEU reagent. After drying, the chromatogram was visualized at UV light (366 nm). The different samples of *D. villosa* root (*n* = 10) were evaluated in triplicate, and the sequence of the zones present in the chromatograms obtained from the samples and the standard were registered. The retention factor (R_f_) of each zone was also calculated.

#### 4.4.4. Quantification of Secondary Metabolites by Spectrophotometry

The quantification of the total phenolic (TPC) (assay A) and the hydrolysable tannins (THC) (assay B) contents present in the extracts of *D. villosa* root was made by spectrophotometry (UV-spectrophotometry, Hitachi U-2000 UV-Vis spectrophotometer, Tokyo, Japan) based on a gallic acid standard calibration curve [100–1200 mg/L for assay A; y = 0.0158x + 0.064, R^2^ =0.999] or [100–800 mg/L for assay B; y = 0.0014x + 0.034, R^2^ = 0.999]. The assays methods used were the followings:

Assay A-Folin-Ciocalteu method: 0.4 mL of the sample was combined and mixed with 2 mL of the Folin-Ciocalteu reagent (diluted with water 1:10 *v*/*v* before use), and the mixture was then added about 1.6 mL sodium carbonate (75 g/L). After 2 h of incubation at room temperature, the absorbance of the mixture was measured spectrophotometrically at 765 nm against the blank reading [48].

Assay B-Method determination of the concentration of the red intermediate: 1.5 mL of a saturated potassium iodate solution was added to a mixture of 1 mL of the extract and 2.5 mL of aqueous acetone 20% (*v*/*v*). After 40 min of incubation at 0 °C, the concentration of the red intermediate originated was measured spectrophotometrically at 550 nm [38].

The TPC and THC value of samples was determined using a gallic acid standard calibration curve, as gallic acid equivalents in milligrams (GAE) per g of the *D. villosa* root dried extract (mg GAE/g DVR).

#### 4.4.5. Analysis of Loss on Drying and Total Ash

The loss on drying and total ash contents were obtained from samples analyzed according to the standard method described in European Pharmacopoeia 10.0 [42,43].

#### 4.4.6. Statistical and Data Analysis

The results were expressed as the mean value ± standard deviation (SD) of the mean. Statistical data analysis was conducted using the independent-samples *t*-test (SPSS Statistics, version 28.0.1.0, 2021, IBM, Armonk, NY, USA), and GraphPad Prism 5.0 statistical software. Statistical significance was found for *p* < 0.05.

## 5. Conclusions

In the present work, it was possible to confirm the usefulness of macro- and microscopic botanical diagnosis methods in the identification of *D. villosa* root as a herbal drug. Preliminary existing data were confirmed, and additional data were obtained to establish the monographic requirements for the correct diagnosis of this medicinal plant. In addition, new data on its chemical composition, particularly concerning its major secondary metabolites, are also presented, as well as data on the granulometry of the powdered raw plant material and of the raw plant material and solvent extraction ratio used. This is of extreme utility as a guarantor of the reproducibility of the chemical composition of future extracts to be prepared with *D. villosa* root for the continuation of chemical, pharmacological and toxicological and pharmacotechnical studies already underway with this medicinal plant with the aim of enabling the development of traditional preparations based on it with the quality, safety, and efficacy required, following the international standards in force.

## Figures and Tables

**Table 1:** *D. villosa*, root–organoleptic features, and size of samples.

Outer Color	Inner Color	Odor	Taste	Average ± SD	
				Length Fracture (cm)	Diameter (cm)
dark brown to black	light brown to orange	earthy and aromatic	bitter and astringent	13.9 ± 0.4	5.2 ± 1.4

**Table 2:** *D. villosa* root micromorphological quantitative analysis.

Botanical Marker	Min *-Max ** μm^2^ × 10^3^	Average ± SD μm^2^ × 10^3^
Sclereids	3.59–29.60	16.59 ± 5.47
Brachysclereids	13.82–442.14	229.15 ± 118.13
Medullary rays	11.07–57.38	24.42 ± 0.98
Xylem Vessels single	2.99–181.80	40.19 ± 40.62
Grouped Xylem Vessels	2.71–42.52	14.19 ± 11.34
Xylem Vessels double	6.20–181.17	32.52 ± 35.51
Prismatic Calcium Oxalate Crystals	0.13–51.09	4.14 ± 9.95
Starch grains	0.19–0.49	0.35 ± 0.07

Results are presented as average ± SD (*n* = 30), about Area μm^2^ × 10^3^, of three independent experiments to a confidence level of 95% (*p* < 0.05). * Min, minimum; ** Max, maximum; SD, standard deviation.

**Table 3:** *D. villosa* root–histochemical screening results.

Compounds	Reagent	Observed Color	Results
Quinones	KOH	Purple/violet	++
Polyphenols	Potassium dichromate	Dark-brown	++
Terpenoids	2,4-dinitrophenylhydrazine, sulphuric anisaldehyde and Liebermann-Burchard	Brown/Orange-red	+
Starch	Lugol’s solution	Brown/Purple	++
Mucilage	Ruthenium red	Red	-
Lipids	Sudan Black III	Orange/Red	-

(++) Presence of compound; (+) weak presence of compound; (-) compound family not found.

**Table 4:** *D. villosa* root, powdered-particle size distribution.

Parameters	GMTM (mm)	SR/T (g)
GM	0.249	6.1
D25	0.117	6.4
D75	0.552	11.4
IQR	0.435	5.1
D50	0.255	10.3
	**Total**	100.0

GM: geometric mean; GMTM: geometric mean of the sieve mesh; SR: sample retained; T: sieve.

**Table 5:** Effect of *D. villosa* root powder particle size on the extract yield.

Sample	45 μm	0.355 mm	1 mm	TC
Dried root (g)	10	10	10	10
Dried extract (g)	1.25	1.36	1.27	1.57
Yield (%)	12.5	13.6	12.7	15.7
**DER (*w*/*w*)**	**7.87:1**	**7.35:1**	**8.00:1**	**6.35:1**

DER: drug-extract ratio; TC: total composition (0.053 mm to 1.086 mm).

**Table 6:** *D. villosa* root-quantitative determination of total phenols, and hydrolyzable tannins.

Secondary Metabolites	Standard Curve Equation (GA)	Total Content (mg GAE/g DVR)
Total Phenolic	y = 0.0158x + 0.064, R^2^ = 0.999	12.33 ± 0.002
Hydrolysable Tannins	y = 0.0014x + 0.034, R^2^ = 0.999	212.29 ± 0.005

Data presented as mean ± SD (*n* = 3), to a confidence level of 95% (*p* < 0.05). GA: gallic acid; GAE: gallic acid equivalent; y: absorbance and x: mg of GAE per g of DVR dried extract.

## Data Availability

Not applicable.
